# Posthospitalization Follow-Up of Patients With Heart Failure Using eHealth Solutions: Restricted Systematic Review

**DOI:** 10.2196/32946

**Published:** 2022-02-15

**Authors:** Ingvild Margreta Morken, Marianne Storm, Jon Arne Søreide, Kristin Hjorthaug Urstad, Bjørg Karlsen, Anne Marie Lunde Husebø

**Affiliations:** 1 Department of Quality and Health Technologies University of Stavanger Stavanger Norway; 2 Research Group for Nursing and Health Sciences Stavanger University Hospital Stavanger Norway; 3 Department of Public Health University of Stavanger Stavanger Norway; 4 Faculty of Health Sciences and Social Care Molde University College Molde Norway; 5 Department of Gastrointestinal Surgery Stavanger University Hospital Stavanger Norway; 6 Department of Clinical Medicine University of Bergen Bergen Norway; 7 Faculty of Health Studies VID Specialized University Oslo Norway

**Keywords:** adherence, eHealth, heart failure, posthospitalization follow-up, patient outcome, review

## Abstract

**Background:**

Heart failure (HF) is a clinical syndrome with high incidence rates, a substantial symptom and treatment burden, and a significant risk of readmission within 30 days after hospitalization. The COVID-19 pandemic has revealed the significance of using eHealth interventions to follow up on the care needs of patients with HF to support self-care, increase quality of life (QoL), and reduce readmission rates during the transition between hospital and home.

**Objective:**

The aims of this review are to summarize research on the content and delivery modes of HF posthospitalization eHealth interventions, explore patient adherence to the interventions, and examine the effects on the patient outcomes of self-care, QoL, and readmissions.

**Methods:**

A restricted systematic review study design was used. Literature searches and reviews followed the (PRISMA-S) Preferred Reporting Items for Systematic Reviews and Meta-Analyses literature search extension checklist, and the CINAHL, MEDLINE, Embase, and Cochrane Library databases were searched for studies published between 2015 and 2020. The review process involved 3 groups of researchers working in pairs. The Mixed Methods Appraisal Tool was used to assess the included studies’ methodological quality. A thematic analysis method was used to analyze data extracted from the studies.

**Results:**

A total of 18 studies were examined in this review. The studies were published between 2015 and 2019, with 56% (10/18) of them published in the United States. Of the 18 studies, 16 (89%) were randomized controlled trials, and 14 (78%) recruited patients upon hospital discharge to eHealth interventions lasting from 14 days to 12 months. The studies involved structured telephone calls, interactive voice response, and telemonitoring and included elements of patient education, counseling, social and emotional support, and self-monitoring of symptoms and vital signs. Of the 18 studies, 11 (61%) provided information on patient adherence, and the adherence levels were 72%-99%. When used for posthospitalization follow-up of patients with HF, eHealth interventions can positively affect QoL, whereas its impact is less evident for self-care and readmissions.

**Conclusions:**

This review suggests that patients with HF should receive prompt follow-up after hospitalization and eHealth interventions have the potential to improve these patients’ QoL. Patient adherence in eHealth follow-up trials shows promise for successful future interventions and adherence research. Further studies are warranted to examine the effects of eHealth interventions on self-care and readmissions among patients with HF.

## Introduction

### Background

Heart failure (HF) affects an estimated 64 million people worldwide [1]. It poses a burden on the health care system in general and on primary care specifically because the total number of patients with HF is increasing, reflecting the chronic course of the disease as well as population growth and aging [2,3]. Symptomatic HF is a complex clinical syndrome with a symptom burden of dyspnea and fatigue [4] and can be troublesome for patients and their families because of frequent hospitalizations and symptom and treatment burden negatively affecting their quality of life (QoL) [5-7]. QoL is understood as a multidimensional and subjective concept that includes physical, functional, emotional, and social well-being [8]. Effective self-care behavior is essential for patients with HF [9,10]. Self-care in the context of HF is an overarching concept based on three key concepts: (1) self-care maintenance (eg, compliance with medication regimens and following diet and physical activity recommendations), (2) self-care monitoring (eg, regular weighing), and (3) self-care management (eg, changing diuretic dose in response to symptoms) [10]. Upon discharge from the hospital, many patients transition from care provided by health professionals in a safe hospital setting to individual self-care at home [11]. This period, when patients transition between hospital and home, is a vulnerable and stressful time for patients with HF and many struggle to perform recommended self-care and navigate the health care system, particularly when posthospitalization care is poorly executed as a result of inadequate coordination of resources or follow-up [5,7]. Of any diagnosis, HF is associated with the highest 30-day all-cause readmission rate (approximately 20%), whereas nearly 35% of the patients with HF are readmitted within 90 days [2,6]. During this phase, the lack of resources for following up or poor medical education leaves this population vulnerable to deterioration and rehospitalization [12]. Posthospitalization HF disease management programs include education, self-management, weight monitoring, sodium restriction or dietary advice, exercise recommendations, and medication review [13]. In addition to social and psychological support with a high degree of care coordination, as well as the higher intensity of follow-up, these components may be important for better self-care behavior, increased QoL, and reduced readmission rates [4,13,14]. The impact of the COVID-19 pandemic has raised the requirement for, and importance of, eHealth solutions as a tool for health care professionals to perform such follow-up of patients with HF [15]. Insight into ensuring a more seamless eHealth care service from inpatient to outpatient care for patients with HF is necessary if they are to achieve adequate self-care support and feel safe [15,16]. eHealth care service is defined as “health services and information delivered or enhanced through the internet and related technologies” [17] and holds the potential to increase the efficiency and quality of health care services [18]. In this review, eHealth comprises digital solutions to deliver health care services, including patient education; telemonitoring of weight, blood pressure, and heart rhythm; and social and emotional support. Previous research suggests that the use of posthospitalization eHealth interventions to follow up on patients may promote self-care for people with long-term illness [18]. Several recent reviews have summarized the findings from eHealth follow-up interventions for patients with HF and provided information about the efficiency of such interventions. Auener et al [19] investigated the effects of telemonitoring programs on different aspects of health care use from 16 randomized controlled trials (RCTs) and 13 nonrandomized studies. All studies included weight as a parameter, whereas only 4 included electrocardiography measures as a physiological parameter. The results revealed that telemonitoring has the potential to reduce hospitalization rates. However, the number of non–emergency department visits increased in most of the studies [18]. Ding et al [20] extracted 18 telemonitoring strategies from 26 RCTs involving patients with HF. Some strategies were commonly used, such as call center support and daily weight monitoring, whereas others, including nurse support, interventions for depression and anxiety, and exercise interventions, were seldom used. Telemonitoring strategies involving medication support and mobile health (mHealth) interventions were associated with improvements in all-cause mortality or hospitalization outcomes [20]. A systematic review conducted in 2017 identified 39 relevant RCTs of telemedicine, largely based on assessments of symptoms, weight, heart rate and rhythm, and blood pressure, and found that telemonitoring was associated with reductions in all-cause mortality of 20% and HF hospitalization of 37% [21]. In contrast, nurse-based telephone-supported care seemed to provide little benefit, and only a reduction in the rate of HF-related admission was noted compared with the control group. However, a combination of home-based teletransmission and nurse-based telephone reinforcement may be encouraged [21]. Although these reviews generally support the effectiveness of eHealth interventions for patients with HF, the outcomes mainly focus on readmission and health care use, and only one of them focuses specifically on the hospital-to-home transition phase [21]. Moreover, they mostly lack information about self-management, QoL, and participants’ adherence to the eHealth interventions. Adherence to self-management and medication regimens is crucial during the transition from hospital discharge to home to prevent hospital readmission and achieve improved health outcomes and QoL [22-24]. Therefore, the success of an intervention aiming to support patients’ chronic disease management depends on patient adherence to the intervention components [25]. Intervention adherence refers to the degree to which the behavior of trial participants corresponds to the intervention assigned to them [26]. Adherence varies according to the patient’s health status, treatment regimens, access to support, and psychological factors such as motivation and beliefs. The long-term success of interventions depends on patients assuming responsibility for their own health and can be achieved with the aid of coordinated measures such as patient education and regular follow-up contacts [26]. An accurate assessment of intervention adherence is warranted to verify whether changes in health outcomes are due to a particular intervention [26].

There is a knowledge gap concerning the synthesis of recent posthospitalization eHealth follow-up interventions for patients with HF focusing on outcomes of self-care, QoL, and adherence to the interventions. Therefore, this restricted review will investigate eHealth interventions that may better prepare patients for the period after hospital discharge, strengthen their self-care and QoL, reduce readmissions, and help them to recover well. Furthermore, the review will address the issue of adherence and discuss how it may affect intervention outcomes. Therefore, the aim is to summarize the most recent information about the content and delivery mode of HF posthospitalization eHealth interventions, explore patient adherence to the interventions, and systematically investigate the effects on patient outcomes of self-care, QoL, and readmissions.

### Research Questions

The a priori research questions were designed according to the FINER framework, which states that a review research question should be feasible, interesting, novel, ethical, and relevant [27].

Our research questions were as follows:

- What are the content and delivery modes of posthospitalization eHealth interventions for patients with HF?
- What is the reported adherence to posthospitalization eHealth interventions in HF?
- Which effects can be expected from posthospitalization eHealth interventions on self-care, QoL, and readmissions of patients who have received treatment for HF?

## Methods

### Reporting Standards

This study used a framework proposed for restricted systematic reviews [27]. The restricted systematic review framework is proposed to be applicable when conducting a rapid review because it consists of core steps that are minimum requirements for systematic reviews, thereby accommodating factors such as a short time frame and limited resources [28]. Such factors are important to consider when conducting a literature search and review as part of developing complex interventions [29]. The framework comprises six core steps: (1) literature search, (2) study selection, (3) data extraction, (4) critical assessment of the included studies, (5) data synthesis, and (6) publication [28].

### Step 1: Literature Search and Search Terms

The literature search was performed as part of a more extensive review study on eHealth interventions in noncommunicable diseases. This paper reports the results from HF populations. A research librarian performed comprehensive literature searches in the CINAHL, MEDLINE, Embase, and Cochrane Library databases. To ensure that our results reflect current conditions and avoid repeating previous review efforts, this rapid review was limited to data published between 2015 and 2020 in English or a Scandinavian language. Searches were performed in the publication title or abstract. Appropriate search terms, including relevant Medical Subject Headings, were closely matched with the Population, Intervention, Control, and Outcome elements (see next section). Documentation of the search strategy and search terms is presented in Multimedia Appendix 1. The search strategy also included manually hand searching the reference lists of the included studies and relevant background material. The searches were performed on March 20, 2020.

### A Priori Eligibility Criteria

Key components of the synthesis are encapsulated by the Population, Intervention, Control, and Outcome framework [30].

- Population: patients initially treated for HF
- Intervention: posthospitalization eHealth follow-up services
- Control: standard care and nondigital follow-up services
- Outcomes: self-management and self-care, QoL, and readmissions

The inclusion and exclusion criteria are displayed in Textbox 1.

**Inclusion and exclusion criteria.**

**Inclusion criteria**

- Empirical intervention studies
- Populations of adult patients with heart failure
- eHealth interventions from hospital to home
- Patient outcomes of self-care, quality of life, and readmissions
- Experimental and quasi-experimental randomized and nonrandomized controlled trials
- Pre–post design with a comparison group
- Peer-reviewed studies
- Published in English

**Exclusion criteria**

- Review studies, study protocols, book chapters, and conference contributions
- Children and adolescent patients
- Older adults (aged >80 years)
- Community health care services context
- >3 months since hospital discharge
- Insufficient detail provided to estimate study outcome
- Mixed patient samples
- Noncomparator study designs

### Step 2: Study Selection

**Figure 1:**
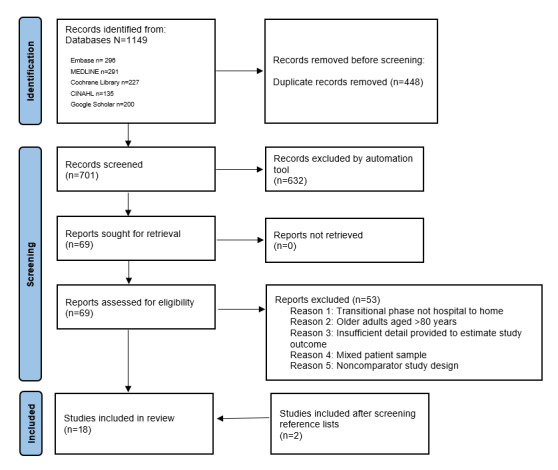
PRISMA (Preferred Reporting Items for Systematic Reviews and Meta-Analyses) flow chart of the study selection process.

After removing duplicates using EndNote (Clarivate), a member of the research team (AMLH) carried out an initial broad review of all included titles and abstracts, using the a priori inclusion and exclusion criteria. Next, the abstracts verified for potential inclusion were reviewed for full-text extraction by all authors, divided into 3 review teams. Full-text articles were extracted for 9.8% (69/701) of the abstracts. Finally, team members resolved conflicting opinions by assessing reasons for exclusion and deciding whether to include the study. The results of the data search and selection process are displayed in a PRISMA (Preferred Reporting Items for Systematic Reviews and Meta-Analyses) flowchart (Figure 1) [31].

### Step 3: Data Extraction

An Excel spreadsheet (Microsoft Corp) was created to ensure consistent data extraction, including data fields of publication identifiers, study design, study context and participants, eHealth intervention or program, and outcomes. The review teams used the spreadsheet to extract relevant data from the included articles. Any inconsistency within the group was resolved through assessment by a reviewer from one of the other groups.

### Step 4: Critical Assessment of Included Studies

To minimize bias, an assessment of internal validity of the included studies, risk of bias (eg, over- or underestimation of intervention effect), and potential conflicts of interest were examined using the Mixed Methods Appraisal Tool (MMAT) [32]. The MMAT, which aims to appraise the methodological quality of included studies in systematic reviews, consists of a checklist of qualitative, quantitative, and mixed methods studies [32]. For this review, checklists for randomized and nonrandomized research designs were used. Each checklist is initiated with 2 screening questions to allow for further assessment, and each list contains 5 assessment criteria to be answered with *Yes*, *No*, or *Can’t tell*. A total score of 7 constitutes a *Yes* response to the screening and assessment criteria [32]. The developers recommend that the MMAT be used to describe only the study quality and to avoid excluding studies based on total scores [32].

To assess data quality, each review team member independently rated the studies, followed by a discussion to achieve consensus. For 10% (2/18) of the included studies, the quality scoring was verified through independent scoring by 2 reviewers (IMM and AMLH). The quality of included studies was above moderate (ie, of the 7 criteria, 6 [86%] were answered with *Yes*; Textbox 1).

### Step 5: Data Synthesis

The findings on service content and delivery mode, adherence, and the effects of posthospitalization follow-up eHealth interventions were systematically analyzed by using thematic analysis as well as searching for patterns, themes, and categories across studies, which were then narratively summarized as suggested by Whittemore and Knafl [33]. Because of the heterogeneity of the study designs, participants, and outcome measures, meta-analysis was not recommended. Thus, the effects on patient outcomes were reviewed and reported narratively.

### Step 6: Publication

The results from this restrictive systematic review will be published, including all appendices and added data. In addition, the study’s findings will be disseminated in relevant clinical settings and websites.

## Results

### Overview

The literature search process is outlined in Figure 1. The search yielded a total of 1149 references (ie, records screened for 2 patient populations); after the removal of 318 (27.68%) duplicates, 831 (72.32%) titles and abstracts were assessed for inclusion. Of the 831 titles and abstracts, 701 (84.4%) titles pertaining to eHealth interventions for patients with HF were screened for eligibility using the web tool [34]. Of the 69 studies evaluated for eligibility in full text, 16 (23%) met all inclusion criteria and were included. Screening the reference lists of the included studies yielded another study and screening the reference lists of relevant background material identified a further study. Finally, this review included 18 studies.

### Study Characteristics

Detailed characteristics of the included studies are displayed in Table 1. All studies were published between 2015 and 2019. Of the 18 studies, 10 (56%) were performed in the United States [35-44]. Although RCT was the predominant study design, 11% (2/18) of the studies applied a quasi-experimental method [37,43]. Among the 18 studies, enrollment of patients with HF to the posthospitalization eHealth service varied from recruitment at the hospital before hospital discharge to recruitment within 3 months of recent hospitalization (Textbox 1). In 56% (10/18) of the studies, all patients were recruited upon hospital discharge to an intervention with a duration of 14 days to 12 months [35,37,38,40-42,44-47]. In 22% (4/18) of the studies, patients were recruited after recent (within 30 days) hospitalization to an intervention with a 3- to 12-month duration [39,43,48,49]. In another study, patients with HF were enrolled during hospitalization or within 3 months of discharge for an HF exacerbation, and the intervention lasted for 3 months [36]. In 17% (3/18) of the studies, patients were recruited at hospital discharge or at the HF outpatient clinic to an intervention with a duration of 3-9 months [50-52].

**Table 1:** Characteristics of included eHealth intervention studies involving patients with heart failure (HF; N=18).

Study (country)	Design	Sample size			Content, focus, and mode of instruction	Duration	MMAT^a^ scores out of 7, n (%)
		Total sample (N)	I^b^, n (%)	C^c^, n (%)			
Athilingam et al [35] (United States)	RCT^d^	18	9 (50)	9 (50)	Telemonitoring (HeartMapp); daily measures of weight, heart rate, blood pressure, and HF symptoms. HF education: 10 modules, home visit after 2-3 days by a nurse. A phone call to all participants. Nurses checked the dashboard daily to monitor participants’ progress.	30 days	2 (29)
Comin-Colet et al [45] (Spain)	RCT	178	81 (45.5)	97 (54.5)	Telemonitoring and telephone support. Daily measures of weight, heart rate, and blood pressure. HF nurses reviewed alarms and alerts from the system every day.	6 months	6 (86)
Dunbar [36] (United States)	RCT	134	70 (52.2)	64 (47.8)	Telephone support; education and counseling on diet, medications, self-monitoring, symptoms, and physical activity; self-monitored blood glucose level and weight; self-care with follow-up home visits and telephone counseling.	6 months	4 (57)
Evangelista et al [37] (United States)	Quasi-experimental	42	21 (50)	21 (50)	Telemonitoring and telephone support; daily measures of weight, heart rate, and blood pressure. Telemonitoring provided alerts and feedback in the case of worrisome responses to questions or if vital signs were outside of preset limits. The research nurse communicated with the patient through teleconferencing and collaborated with the patient’s primary care provider to facilitate a plan of action. Telephone support as usual to the control group.	3 months	7 (100)
Frederix et al [46] (Belgium)	RCT	160	80 (50)	80 (50)	Telemonitoring; daily measurements of weight, heart rate, and blood pressure were forwarded to a central computer. If the recordings were outside of predefined alert limits, both the general practitioner and HF clinic were alerted by email. At that moment, per protocol, the general practitioner (or cardiologist) was asked to visit or contact the patient and adapt the treatment if they felt that it was necessary. The HF nurse contacted the patient by telephone 1-3 days after the alert to verify whether the intervention had been effective.	6 months	6 (86)
Gallagher et al [38] (United States)	RCT	40	20 (50)	20 (50)	Telemonitoring; electronic measurement of adherence to loop diuretics. A licensed clinical social worker reviewed adherence data daily during the first 7 days after discharge and weekly after that and then contacted participants who were nonadherent for ≥2 days per week.	30 days	7 (100)
Hwang et al [48] (Australia)	RCT	53	24 (45.3)	29 (54.7)	Telemonitoring and telephone support; participants were instructed to self-monitor and verbally report their blood pressure, heart rate, and oxygen saturation levels at the start of each rehabilitation session. The intervention group received electronic education sessions.	3 months	7 (100)
Jayaram et al [39] (United States)	RCT	1521	756 (49.7)	765 (50.3)	Telephone calls are used for technical support by interactive voice response; symptoms and daily weight; patients were instructed to call a toll-free number daily for 6 months, respond to a series of automated questions regarding their symptoms, and enter their daily weight. They were also provided with educational materials.	6 months	6 (86)
Kotooka et al [47] (Japan)	RCT	181	90 (50)	91 (50)	Telemonitoring and telephone support, measurement of weight, heart rate, and blood pressure daily. Physicians could provide telephone guidance, change medications, and order hospital readmission if required. Full-time nurses monitored acquired data on a secure website. Telephone support from a physician as usual.	15 months	6 (86)
Kraai et al [50] (Netherlands)	RCT	176	83 (47.2)	93 (52.8)	Telemonitoring and telephone support; daily measurement of weight, heart rate, and blood pressure. HF nurses automatically received notifications by mobile phone and email and then discussed symptoms and treatment with patients within 2 hours.	9 months	6 (86)
Köberich et al [51] (Germany)	RCT	110	58 (52.7)	52 (47.3)	Telephone support; nurse-led symptom monitoring, education on signs and symptoms of worsening HF, HF-specific diet, and fluid restriction. When seeking help, patients were advised to use a diary to document body weight, blood pressure, heart rate, and edema on a daily basis. If necessary, after discharge from the hospital, patients received 4 telephone calls within 3 months about changes in HF-related symptoms and treatment.	3 months	5 (71)
Lycholip et al [49] (Netherlands)	RCT	118	58 (49.2)	60 (50.8)	Telemonitoring and telephone support; daily measurement of body weight, blood pressure, and heart rate. HF nurses automatically received notifications by mobile phone and email and, within 2 hours, discussed the symptoms and treatment with the patients. An HF nurse provided education on HF.	9 months	6 (86)
Masterson- Creber et al [40] (United States)	RCT	67	41 (61.2)	26 (38.8)	Telephone support MI^e^: a tailored intervention at discharge to improve self-care, involving a home visit and follow-up calls. A nurse used the MI approach to identify client-directed self-care goals. Participants received written educational material.	3 months	6 (86)
Ong et al [41] (United States)	RCT	1437	715 (49.7)	722 (50.3)	Telemonitoring and telephone support; weight, heart rate, and blood pressure were measured daily. A total of 9 telephone health coaching calls over 6 months, generally from the same call center nurse.	6 months	5 (71)
Pedone et al [52] (Italy)	RCT	96	50 (52/1)	46 (47.9)	Telemonitoring and telephone support; measurement of blood pressure, oxygen saturation, weight, and heart rate daily; a geriatrician evaluated the data received every day. Participants received education on medical treatment and lifestyle counseling by telephone.	6 months	6 (86)
Ritchie et al [42] (United States)	RCT	511	253 (49.5)	258 (50.5)	Interactive voice response and telephone support; symptoms and body weight measured daily; E-Coach intervention: an intervention with condition-specific customization and in-hospital and postdischarge support by a care transition nurse, interactive voice response, postdischarge calls, and care transition nurse follow-up.	2 months	7 (100)
Srivastava et al [43] (United States)	Cohort–control	1067	197 (18.5)	870 (81.5)	Telemonitoring and telephone support; measurement of heart rate and blood pressure daily. Data were monitored on weekdays by a telehealth nurse who analyzed the data for abnormalities and lack of response; if clinical data caused concern for declining health status, a phone call was initiated to the patient. All patients also received a monthly follow-up call.	12 months	6 (86)
Young et al [44] (United States)	RCT	105	54 (51.4)	51 (48.6)	Telephone support: the patient-activated care at home intervention contained a variety of formats (eg, verbal, written, and visual) with 12 weeks of post discharge education sessions delivered by telephone. Besides self-management workbooks, each subject was provided with a self-management toolkit, including a calendar for weight and daily salt-intake logging, a step-on weight scale with large and bright readings, and an electronic pill organizer reminder alarm.	6 months	6 (86)

^a^MMAT: Mixed Methods Appraisal Tool.

^b^I: intervention.

^c^C: control.

^d^RCT: randomized controlled trial.

^e^MI: motivational interview.

### Themes Derived From Data Analysis

In the following section, the data analysis results are presented, thereby answering the research questions concerning intervention content and delivery mode, intervention adherence, and the effects of eHealth on patient outcomes.

### Delivery Mode and Content of Posthospitalization eHealth Follow-Up Interventions

In all, 2 different modes of delivering an eHealth service were identified (Table 1). The specific technologies identified included (1) structured telephone calls and (2) telemonitoring or telemonitoring in combination with telephone support.

#### Structured Telephone Call

Of the 18 studies in our review, 6 (33%) included structured telephone calls to deliver the intervention to patients with HF [36,39,40,42,44,51]. Of these 6 studies, in 2 (33%), interactive voice response devices were used to examine the patients’ symptoms and vital sign registrations [39,42]. In these studies, patients were instructed to call a toll-free number daily for 6 months, respond to a series of automated questions about their symptoms, and enter their daily weight. Responses that met prespecified criteria triggered a variance within the system. Conflicts were then flagged for immediate attention by on-site clinicians [39].

Nurses performed all telephone calls. Four dominant categories of content and use of the telephone-supported HF interventions were identified as follows: (1) keeping logs: encouraging patients to keep logs for monitoring symptoms, blood pressure, and weight; (2) goal-setting skills: teaching patients goal-setting skills to manage their condition or behavior changes; (3) problem-solving skills: teaching patients problem-solving skills to manage their condition; and (4) advice about when to seek help in case of worsening HF. In addition, education and counseling were combined with follow-up home visits in 17% (1/6) of the studies [36], whereas in another study, customized HF education was provided on the patient’s response to questions on symptoms and self-management [40]. Each intervention session lasted 15-50 minutes. In the trial conducted by Ritchie et al [42], support calls were provided to patients only when required, whereas in 67% (4/6) of the studies, 4-10 calls were delivered for 2-4 months [36,40,44,51].

#### Telemonitoring

Of the 18 included studies, 12 (67%) included a telemonitoring program. In 75% (9/12) of these studies, weight, heart rate, and blood pressure were measured daily [35,37,41,45-47,49,50,52]. Athilingam et al [35] also included a medication tracker in their HeartMapp app and physiological exercises to reset the autonomic nervous system and improve functional capacity. Pedone et al [52] included oxygen saturation in addition to measuring blood pressure and heart rate daily. These studies also used assessments of symptoms related to HF and action plans for clinical decisions based on out-of-limit alerts from the data monitoring. In 75% (9/12) of the studies, nurses specialized in HF care and telemedicine, or care transition performed the daily data monitoring [35,37,41,43,45-47,49,50]. Of the 12 telemonitoring studies, 2 (17%) provided patients with automated feedback triggered by out-of-limit alerts [35,39]. In cases where these alerts indicated possible mild to moderate decompensation, nurses could promote diuretic dose adjustments following specific protocols [45] and alerts could be routed to clinicians (eg, physicians and cardiologists) who evaluated the data and contacted patients if necessary. For cases in which out-of-limit alerts indicated severe decompensation, patients were advised to call the emergency number or go to the nearest hospital emergency department [35,37,41,43,45-48,50]. In 17% (2/12) of the studies, clinicians (physicians and geriatricians) conducted data monitoring and management simultaneously [39,52]. Of the 12 studies, 1 (8%) was a telerehabilitation investigation in which participants were guided to self-monitor and verbally report their blood pressure, heart rate, and oxygen saturation levels at the start of each rehabilitation session [48]. Finally, 75% (9/12) of the telemonitoring studies provided the participants with telephone support to either follow up on alerts generated from the patient’s registrations of symptoms and vital signs [43,45,49,50], technical support [48], and follow-up of control group or as usual care [37,47] or to provide patient education [41,52].

### Adherence to Posthospitalization Follow-Up eHealth Interventions in HF

Of the 18 included studies, 11 (61%) reported patients’ adherence to the intervention [35,38,41,42,44,45,47-50,52]. In 91% (10/11) of these studies, adherence was reported as a secondary study outcome, whereas in 9% (1/11) of the studies, adherence was included as a primary outcome [38]. Overall, adherence levels were reported at a rate of 72%-99%. Among the studies using telephone support as a delivery mode, 33% (2/6) included measures of adherence with adherence levels of 86% [42] and 84% (T1) and 86% (T2) [44]. Details regarding reported adherence are provided in Table 2.

**Table 2:** Reporting intervention program adherence in the included studies (N=18).

Study	Adherence reported	Definition and assessment of adherence	Adherence results
Athilingam et al [35]	Yes	Duration for which the participants accessed intervention features.	Adherence was low, with only 72% of the participants completing the 30-day follow-up.
Comin-Colet et al [45]	Yes	Daily automated telemonitoring of biometrics and symptoms using the intervention platform.	Adherence was very high, with missed biometric daily transmissions less than 1% of the expected number of daily transmissions.
Dunbar et al [36]	No	—^a^	—
Evangelista et al [37]	No	—	—
Frederix et al [46]	No	—	—
Gallagher et al [38]	Yes	Adherence to loop diuretics in the 30 days after discharge. Nonadherence=adherence <88%. Adherence was calculated as the percentage of days on which the correct number of doses was taken as prescribed, irrespective of dose timing.	Median correct dosing adherence was 81%, and 33% of the participants were classified as adherent. Reasons for nonadherence were identified as follows: ran out of pills, out of usual routine, side effects, and did not know the correct dose.
Hwang et al [48]	Yes	Attendance rates were categorized into adherent (>80%), partly adherent (20%-80%), and nonadherent (<20%), based on the proportion of sessions attended by each participant.	Of the 51 participants who attended the rehabilitation programs, 49 (96%) were categorized as adherent or partly adherent. None of the intervention participants were nonadherent.
Jayaram et al [39]	No	—	—
Köberich et al [51]	No	—	—
Kotooka et al [47]	Yes	Adherence was measured as the number of days that each patient measured their body weight and blood pressure in a month.	The mean rates of adherence at 1, 6, and 12 months after randomization were 96%, 90%, and 91%, respectively.
Kraai et al [50]	Yes	Adherence of patients to telemonitoring was assessed by daily weighing and measuring of blood pressure.	The median adherence rate was 95% (range 87%-99% for the total study period).
Lycholip et al [49]^b^	Yes	Adherence of patients to telemonitoring was assessed by daily weighing and measuring of blood pressure.	The median adherence rate was 95% (range 87%-99% for the total study period).
Masterson-Creber et al [40]	No	—	—
Ong et al [41]	Yes	Telemonitoring adherence: percentage of total days during 30 and 180 days; telephone coaching adherence: percentage of total days during 30 and 180 days.	Overall, 83% (591/715) of the intervention participants used telemonitoring equipment.
Pedone et al [52]	Yes	Percentage of the total amount of expected symptom measurements.	On average, 62% of the scheduled measurements were completed (weight once a day, blood pressure and heart rate twice a day, and peripheral oxygen saturation thrice a day); adherence was best for pulse oximeter (70%) and worst for the scale (56%); 64% of the participants completed at least half of the scheduled measurements.
Ritchie et al [42]	Yes	Total (100%) adherence was defined as answering all interactive voice response system calls. Optimal adherence: daily response to the interactive voice response during the first 7 days. Answering a call was defined as a patient completing the questions of the call.	Of the patients with HF, 144 (86%) received a total intervention dose.
Srivastava et al [43]	No	—	—
Young et al [44]	Yes	Frequencies of self-reported self-management behaviors of daily weighing, following a low-sodium diet, taking prescribed medications, exercising, and attending follow-up appointments.	Participants in the intervention group who received the patient-activated care at home intervention had significantly higher self-reported adherence to self-management behaviors; 84% at 3 months and 86% at 6 months reported not missing any doses in the previous week, compared with 68% at both time points in the control group.

^a^Data not available.

^b^Same study population and intervention as in the study by Kraai [50].

### Effects From Follow-Up Interventions on Patient Outcomes

#### Overview

Of the 18 included studies, only 1 (6%) investigated all 3 patient outcomes of interest to this review (ie, QoL, readmissions, and self-care behavior) [45]. Included in 61% (11/18) of the studies, QoL was the most frequently analyzed patient outcome, followed by readmissions in 56% (10/18) of the studies. Self-care was explored in 44% (8/18) of the included studies. Details concerning the effects of eHealth interventions are provided in Table 3.

**Table 3:** Effects of intervention programs on patient outcomes of quality of life (QoL), self-care, and readmissions (N=18).

Study	Sample size n (%), I^a^/C^b^	Baseline		Postbaseline measures		Outcome
				T1^c^ (days), P value	T2^d^ (days), P value	
Athilingam et al [35]	9/9 (50/50)		Hospital discharge	.93 (30) .01 (30) .03 (30) .18 (30)	N/A^e^	Self-care maintenance Self-care management Self-care confidence QoL
Comin-Colet et al [45]	81/97 (46/54)		Hospital discharge	.06 (180) .001 (180) .01 (180)	N/A	Self-care QoL Readmissions
Dunbar et al [36]	54/54 (50/50)		Hospital discharge or within 3 months after discharge	<.001 (90)	.002 (180)	QoL
Evangelista et al [37]	21/21 (50/50)		Hospital discharge	<.001 (90) <.001 (90) <.001 (90) <.001 (90) <.001 (90)	N/A	QoL overall QoL emotional subscale Self-care maintenance Self-care management Self-care confidence
Frederix et al [46]	80/80 (50/50)		Hospital discharge	.04 (180)	N/A	Readmissions
Gallagher et al [38]	20/20 (50/50)		Hospital discharge	.41 (30) .72 (30)	N/A	Self-care (medication adherence) Readmissions
Hwang et al [48]	24/26 (48/52)		Recent discharge	.03 (360)	.03 (720)	QoL
Jayaram et al [39]	756/765 (49.7/50.3)		Recent discharge	.32 (90)	.04 (180)	QoL
Kotooka et al [47]	90/91- (50/50)		Hospital discharge	.94 (352) .42 (352)	N/A	QoL HFf readmissions
Kraai et al [50]	94/83 (53/47)		Hospital discharge or outpatient clinic	.62 (270) .87 (270)	N/A	QoL HF readmissions
Köberich et al [51]	58/52 (53/47)		Hospital discharge or outpatient clinic	.20 (90) <.001 (90)	N/A	QoL Self-care
Lycholip et al [49]	58/60 (49/51)		Recent discharge	.77 (90)	N/A	Self-care
Masterson-Creber et al [40]	41/26 (61/39)		Hospital discharge	.36 (90) .03 (90) .31 (90)	N/A	QoL Self-care maintenance Self-care confidence
Ong et al [41]	715/722 (49.8/50.2)		Hospital discharge	.25 (30) .63 (30)	.02 (180) .54 (180)	QoL Readmissions
Pedone et al [52]	50/46 (52/48)		Hospital discharge or outpatient clinic	.04 (180)	N/A	Readmissions
Ritchie et al [42]	245/233 (51.3/48.7)		Hospital discharge	.18 (30)	N/A	Readmissions
Srivastava et al [43]	197/870 (18.5/81.5)		Recent discharge	.07 (352)	N/A	Readmissions
Young et al [44]	54/51 (51/49)		Hospital discharge	.09 (90) <.001 (90)	.09 (180) <.001 (180)	Readmissions Self-care adherence

^a^I: intervention.

^b^C: control.

^c^T1: first postbaseline data collection.

^d^T2: second postbaseline data collection.

^e^N/A: not applicable.

^f^HF: heart failure.

#### Impact on QoL

QoL was included as a patient outcome in 61% (11/18) of the studies among patients with HF [35-37,39-41,45,47,48,50,51]. Of these 11 studies, 4 (36%) found that an eHealth intervention significantly improved patients’ *overall QoL* [36,37,45,48]. Of these 4 studies, 3 (75%) contained telemonitoring combined with telephone support and with an intervention duration of 3-6 months [35,43,45], whereas the study by Dunbar et al [36] provided only telephone support lasting for 6 months. In both the studies by Jayaram et al [39] and Ong et al [41], the QoL was nonsignificant at the first postbaseline data collection. In contrast, QoL was significantly improved in both studies 6 months after beginning the intervention [39,41]. Of the 3 studies recruiting participants later than at discharge (ie, within 30 days after discharge), 2 (67%) reported significant effects from an eHealth intervention on QoL [39,48].

#### Self-care Behavior

Self-care was investigated in 39% (7/18) of the studies [35,37,38,44,45,49,51]. Athilingam et al [35] and Evangelista et al [37] both reported on self-care measured by the Self-Care of Heart Failure Index, which included the subscales self-care maintenance, self-care management, and self-care confidence. *Self-care management* was found to be significantly increased by eHealth interventions in both studies; in addition, Evangelista et al [37] found that *self-management maintenance* also seemed to be significantly improved. The subscale *self-care confidence* was enhanced to a significant degree by an eHealth intervention in 14% (1/7) of the studies [35]. Comin-Colet et al [45] used the Self-care Behavior Scale to study self-care behavior in patients with HF who were remotely followed by the Home Tele-HealthCare platform, with the authors detecting a marginally significant difference between the intervention and control groups. Köberich et al [51] and Lycholip et al [49] measured self-care behavior by using the European Heart Failure Self-care Behavior Scale. Of the 7 studies, 1 (14%) found significant improvements from the eHealth interventions on self-care behavior [51], whereas Lycholip et al [49] determined that such interventions did not influence self-care behavior; this study recruited patients within 14 days after discharge [49]. Finally, in the study by Gallagher et al [38], self-care was defined as medication adherence, with no significant effect from the eHealth intervention being noted. The studies showing significant effects on self-care behavior delivered the interventions for 30 days to 6 months. Of the 7 studies, only 1 (14%) did not include digital monitoring of symptoms and vital signs [46].

#### Readmissions

Of the 18 studies, 10 (56%) included readmissions as a patient outcome [38,41-47,50,52]. A significant reduction in readmissions associated with eHealth interventions was detected in 30% (3/10) of these studies [45,46,52]. All the studies combined telemonitoring and telephone support as the intervention delivery mode, and the intervention lasted for 6 months. Comin-Colet et al [45] found a significant reduction in readmissions in the HF intervention group compared with controls. The study by Frederix et al [46] identified a significant reduction (P=.04) in days lost to HF-related readmissions among patients in the intervention group but not for all-cause readmissions (P=.26). Pedone et al [52] revealed a significantly (P=.04) higher risk of readmissions (42%) at 180 days in the control group compared with 21% for patients with HF who were given remote follow-up. None of the studies that recruited patients later than discharge achieved significant effects on readmissions [43,44,47].

## Discussion

### Summary of Evidence and Comparison With Prior Work

In this restricted systematic review, we have evaluated and synthesized the findings from 18 posthospitalization follow-up eHealth interventions targeting QoL outcomes, self-care, and readmissions of patients with HF. To summarize, patients with HF were enrolled in the interventions upon or after hospital discharge. Interventions were delivered mainly by telephone or email and focused on patient education and counseling, social and emotional support, and self-monitoring of vital signs and symptoms. Posthospitalization eHealth follow-up for patients with HF holds potential for improving their QoL, whereas a positive impact on self-care and readmissions is less evident.

Some of the included studies used more traditional tools to follow up with patients, such as the telephone. Because of its familiarity and ease of use, the telephone may be appropriate to contact patients remotely. Individuals at risk of low eHealth literacy, such as older or less educated patients, may benefit from using a more traditional eHealth tool such as the telephone [53]. However, when comparing the effects on patient outcomes from studies using eHealth solutions other than the telephone as the delivery mode, telephone interventions do not stand out as more or less appropriate. We found that telephone interventions as a delivery mode effectively improved patients’ self-care behaviors, but the effects on QoL and readmissions were less promising. This finding suggests that self-care follow-up is likely to be more important than the specific mode of follow-up.

Most studies in this review included features that required patients to monitor their vital signs and report health behaviors and symptoms. Giving patients with HF a more active role in their healing processes through posthospitalization eHealth interventions may promote their experience as true partners in shared decision-making, improve their well-being, and result in better adherence to treatment [54]. However, the value of eHealth interventions as part of health care for patients who are chronically ill may vary. Runz-Jørgensen et al [55] found that patients with multimorbidity and more significant illness and treatment burden perceived eHealth interventions as more favorable than those with less complex disease and treatment. This result may be explained by considering the burdens of HF [55]. Patients with HF are vulnerable because they require regular and ongoing disease monitoring and management to reduce the risk of deterioration, and many fragile patients with HF have limited access to the health care system. The COVID-19 pandemic has forced health care systems to re-evaluate reimbursement for eHealth solutions to promote more widespread adoption of HF care [15,56,57].

We believe that for patients with HF to perceive the eHealth follow-up service as appropriate and be willing to use it, the timing of the introduction of the service is a crucial factor. In our review, patients were primarily enrolled in the eHealth interventions upon hospital discharge, ensuring patient support immediately after release. However, of the 18 included studies, 5 (28%) recruited patients during the first 4 weeks after discharge, demonstrating that eHealth interventions significantly increased QoL but had little impact on readmissions and self-care. Nevertheless, the findings of the effects from eHealth follow-up on patient outcomes suggest that patients with a severe heart condition benefit from prompt posthospitalization follow-up. It may be essential to provide patients with HF with self-care support at discharge to avoid 30-day readmission.

Remote monitoring as a feature of eHealth interventions may include parameters for detecting symptom and illness deterioration, successfully reducing readmissions among patients with HF [19-21]. However, in our study, the effects on readmissions from remote monitoring were inconclusive. For monitoring to be successful, aspects of measurement reliability and frequency, patient interface and adherence, and prompt interpretation by health professionals need to be considered [58]. Most of the included studies involving remote monitoring also provided contact with health care professionals, mainly nurses, who regularly stayed in touch with patients by either telephone calls or email. Koivunen and Soranto [59] identified communication and patient–nurse relationships as essential factors of telehealth in nursing practice. Patient–nurse interactions enable nurses to inquire about and assess patients’ self-care needs and symptoms, express empathy, and increase patients’ sense of security [58]. Another vital aspect of the patient–nurse interaction in the included eHealth interventions was whether the technology was acceptable to patients. Lack of required engagement among patients may be attributed to the nature of the technology [60], and patient adherence to the system is crucial for an intervention’s success. Ding et al [61] found high adherence to the intervention component of weight monitoring (6 out of 7 days) in their recent telemonitoring RCT of patients with HF (published after our literature research). The intervention resulted in a significant improvement in self-management related to health maintenance, medication adherence, and diet [61]. We found that intervention adherence in most of the remote monitoring studies with patient–nurse interaction was 81%-99%. Comin-Colet et al [62] found that despite low expectations among patients before entering a telemedicine HF care intervention, adherence and satisfaction levels were high during the intervention, likely because of the HF care teams’ proactive engagement with patients [62]. Clinicians who practice patient-centered communication adopting the patient’s perspective may contribute to increased adherence levels in patients with HF, particularly during care transitions such as discharge from hospital to home, which to many patients can be confusing and demanding related to follow-up on treatment regimens [63]. The World Health Organization states that the quality of the treatment relationship is an essential determinant of adherence [26].

eHealth interventions have excellent potential to reinforce patient education on self-care [53]. Most of the reviewed studies in this review provided patients with education or counseling delivered by nurse specialists before the trial or during the trial, covering disease- and treatment-specific topics, psychosocial issues, and health behavior change. These studies seem to support improved self-care from eHealth interventions that include an educational aspect. Although the educational focus of many eHealth and mHealth interventions is illness management [64], a more holistic approach to self-care education not limited to only disease management is suggested. According to Lewis et al [65], addressing the holistic needs of patients with comorbidities using eHealth technology supports more patient-centered health care. Interestingly, of the 18 included studies, only 1 (6%) assessed changes in patients’ knowledge at the completion of the intervention period. This study found that an HF education program involving iterative teaching tools expanded patients’ HF knowledge [35]. This finding is in line with a review by Bashi et al [64], in which only 2 of the 15 mHealth interventions included an evaluation of patient knowledge as a study outcome. On the contrary, a recent Cochrane review of mHealth-delivered educational interventions for patients with HF found no evidence of a difference in HF knowledge or other patient-reported outcomes [66]. However, validated tools of patient knowledge can be an efficient measure of intervention success, and an assessment of patient knowledge as part of eHealth protocols is recommended [66].

### Limitations

Several limitations should be mentioned. First, heterogeneity in the included studies made meta-analysis impossible, and a qualitative thematic analysis was applied. Such an analysis is prone to interpretation bias [33]. Second, the included eHealth interventions pertain to the transition phase between hospital and home, thus limiting generalizability to all stages of follow-up of patients with HF. Third, although most of the included studies indicated good methodological quality, most of them did not apply a blinded randomization process, and 50% (9/18) of the studies did not report adherence to the intervention.

### Conclusions

This review identified 18 studies of posthospitalization follow-up interventions in patients with HF. Most of the included studies enrolled patients in eHealth interventions upon hospital discharge to ensure support in the critical post–hospital discharge period. The most common mode for posthospitalization follow-up was telemonitoring with telephone support. Patients received education or counseling about their disease, psychosocial issues, and health behavior changes. Most studies also required the patients to monitor vital signs and report their health behaviors and symptoms.

The findings of the effects of interventions on patient outcomes such as QoL, self-care, and readmissions propose that patients with HF should receive prompt follow-up after hospital discharge. eHealth interventions, including patient education, support, and self-monitoring, have the potential to improve QoL, but it is less clear how eHealth interventions affect self-care behavior and readmissions in populations of patients with HF.

Aspects of measurement reliability and frequency, user interface and adherence, and prompt interpretation by health professionals need to be considered to ensure successful monitoring in eHealth interventions. These findings are important to inform future intervention studies to support patients with HF after discharge from the hospital. eHealth interventions have the potential to improve well-being, adherence to treatment, and patients’ experiences of being engaged partners in shared decision-making.

Systematic reviews of the literature are recommended during the planning and development of complex interventions [29]. The findings from this review will be used to inform the development of a post–hospital discharge follow-up service addressing the burden of treatment and self-management among patients with HF.

## Appendix

Multimedia Appendix 1 Documentation of the literature search.

## Abbreviations

HF: heart failure
mHealth: mobile health
MMAT: Mixed Methods Appraisal Tool
QoL: quality of life
RCT: randomized controlled trial
