# Supplementary material for: Posthospitalization Follow-Up of Patients With Heart Failure Using eHealth Solutions: Restricted Systematic Review
Source: J Med Internet Res. 2022 Feb 15;24(2):e32946. doi: 10.2196/32946 (PMC8889479; doi:10.2196/32946)
Supplement: Multimedia Appendix 1 [file jmir_v24i2e32946_app1.pdf]

Literature Search - Documentation

**Database/source**	**Embase**
**Date of search**	20.03.2020
**Search History**	1 exp colon tumor/ (315014)<br>2 ((colorectal or colon* or rectal or sigmoid or sigmoid colon or colon sigmoid or mesocolon) adj (cancer* or<br>carcinoma* or neoplasm* or tumor* or tumour*)).ti,ab,kw. (273006)<br>3 1 or 2 (375901)<br>4 exp heart failure/ (488546)<br>5 (heart failure* or heart backward failure* or cardiac failure* or cardiac backward failure* or cardiac<br>incompetence* or cardiac insufficienc* or cardiac stand still or cardial decompensation* or cardial insufficienc* or heart insufficienc* or insufficientia cardis or myocardial failure* or myocardial insufficienc*).ti,ab,kw. (298022)<br>6 4 or 5 (541399)<br>7 3 or 6 (914765)<br>8 exp telehealth/ (42935)<br>9 (telemedicine or teleconsult or telepatholog* or telerehab* or videoconsult* or skype or Mobile Health or ehealth<br>or mhealth or digital health).ti,ab,kw. (28477)<br>10 ((video* or telephone* or tele* or digital or e-mail or email or remote) adj3 (consult* or rehab* or counsel* or<br>therap*)).ti,ab,kw. (11586)<br>11 8 or 9 or 10 (61177)<br>12 7 and 11 (2753)<br>13 self care/ (56957)<br>14 (self care or selfcare or self management or selfmanagement or self treatment or selftreatment).ti,ab,kw. (52150)<br>15 "quality of life"/ (456919)<br>16 ("quality of life" or life quality or HRQL or HRQOL or wellbeing or well-being or well being).ti,ab,kw. (534369)<br>17 (treatment burden* or "burden of treatment*").ti,ab,kw. (3428)<br>18 hospital readmission/ (61075)<br>19 (readmission* or rehospitalization* or rehospitalisation*).ti,ab,kw. (56445)<br>20 13 or 14 or 15 or 16 or 17 or 18 or 19 (785603)<br>21 12 and 20 (1093)<br>22 limit 21 to yr="2015 -Current" (576)<br>23 limit 22 to embase (296)
**Number of results**	296
**Comments**	

Literature Search - Documentation

**Database/source**	**Ovid MEDLINE(R) ALL**
**Date of search**	20.03.2020
**Search History**	1 exp Colorectal Neoplasms/ (197528)<br>2 ((colorectal or colon* or rectal or sigmoid or sigmoid colon or colon sigmoid or mesocolon) adj (cancer* or<br>carcinoma* or neoplasm* or tumor* or tumour*)).ti,ab,kf. (188857)<br>3 1 or 2 (253686)<br>4 exp Heart Failure/ (119088)<br>5 (heart failure* or heart backward failure* or cardiac failure* or cardiac backward failure* or cardiac<br>incompetence* or cardiac insufficienc* or cardiac stand still or cardial decompensation* or cardial insufficienc* or heart insufficienc* or insufficientia cardis or myocardial failure* or myocardial insufficienc*).ti,ab,kf. (183602)<br>6 4 or 5 (212851)<br>7 3 or 6 (466222)<br>8 exp Telemedicine/ (27361)<br>9 (telemedicine or teleconsult or telepatholog* or telerehab* or videoconsult* or skype or Mobile Health or ehealth<br>or mhealth or digital health).ti,ab,kf. (23119)<br>10 ((video* or telephone* or tele* or digital or e-mail or email or remote) adj3 (consult* or rehab* or counsel* or<br>therap*)).ti,ab,kf. (8308)<br>11 8 or 9 or 10 (44301)<br>12 7 and 11 (1239)<br>13 Self Care/ (32616)<br>14 (self care or selfcare or self management or selfmanagement or self treatment or selftreatment).ti,ab,kf. (35892)<br>15 "Quality of Life"/ (189590)<br>16 ("quality of life" or life quality or HRQL or HRQOL or wellbeing or well-being or well being).ti,ab,kf. (349469)<br>17 (treatment burden* or "burden of treatment*").ti,ab,kf. (1852)<br>18 Patient Readmission/ (16631)<br>19 (readmission* or rehospitalization* or rehospitalisation*).ti,ab,kf. (31107)<br>20 13 or 14 or 15 or 16 or 17 or 18 or 19 (481117)<br>21 12 and 20 (512)<br>22 limit 21 to yr="2015 -Current" (291)
**Number of results**	291
**Comments**	

Literature Search - Documentation

Database/source	**Cochrane Library**
Date of search	20.03.2020
Search History	#1 MeSH descriptor: [Colorectal Neoplasms] explode all trees 7862<br><br>#2 ((colorectal or colon* or rectal or sigmoid or "sigmoid colon" or "colon sigmoid" or mesocolon) NEXT (cancer* or<br><br>carcinoma* or neoplasm* or tumor* or tumour*)) 19754<br><br>#3 #1 or #2 19895<br><br>#4 MeSH descriptor: [Heart Failure] explode all trees 9088<br><br>#5 heart NEXT failure* or heart NEXT backward NEXT failure* or cardiac NEXT failure* or cardiac NEXT backward NEXT failure* or cardiac NEXT incompetence* or cardiac NEXT insufficienc* or "cardiac stand still" or cardial NEXT decompensation* or cardial NEXT insufficienc* or heart NEXT insufficienc* or "insufficientia cardis" or myocardial NEXT failure* or myocardial NEXT insufficienc* 29721<br><br>#6 #4 or #5 29737<br><br>#7 #3 or #6 49487<br><br>#8 MeSH descriptor: [Telemedicine] explode all trees 2314<br><br>#9 (telemedicine or teleconsult or telepatholog* or telerehab* or videoconsult* or skype or "Mobile Health" or ehealth or mhealth or "digital health"):ti,ab,kw 6000<br><br>#10 ((video* or telephone* or tele* or digital or "e-mail" or email or remote) NEAR/2 (consult* or rehab* or counsel* or therap*)) 4815<br><br>#11 #8 or #9 or #10 9771<br><br>#12 #7 and #11 541<br><br>#13 MeSH descriptor: [Self Care] this term only 4019<br><br>#14 ("self care" or selfcare or "self management" or selfmanagement or "self treatment" or selftreatment):ti,ab,kw 13545<br><br>#15 MeSH descriptor: [Quality of Life] this term only 22749<br><br>#16 ("quality of life" or "life quality" or HRQL or HRQOL or wellbeing or "well-being" or "well being"):ti,ab,kw 113086<br><br>#17 (treatment NEXT burden* or burden NEXT of NEXT treatment*):ti,ab,kw 365<br><br>#18 MeSH descriptor: [Patient Readmission] this term only 1005<br><br>#19 (readmission* or rehospitalization* or rehospitalisation*):ti,ab,kw 7769<br><br>#20 #13 or #14 or #15 or #16 or #17 or #18 or #19 128019<br><br>#21 #12 and #20 321<br><br>#22 #21 with Cochrane Library publication date from Jan 2015 to present 227
Number of results	227
Comments	

Literature Search - Documentation

**Database/source**	**CINAHL**
**Date of search**	20.03.2020
**Search History**	S22 S12 AND S20 Limiters - Published Date: 20150101-20201231 135<br>Search modes - Boolean/Phrase<br><br>S21 S12 AND S20 Search modes - Boolean/Phrase 274<br><br>S20 S13 OR S14 OR S15 OR S16 OR S17 OR S18 OR S19 Search modes - Boolean/Phrase 319,52<br><br>S19 readmission* or rehospitalization* or rehospitalisation* Search modes - Boolean/Phrase 21,962<br><br>S18 (MH "Readmission") Search modes - Boolean/Phrase 13,944<br><br>S17 "treatment burden*" or "burden of treatment*" Search modes - Boolean/Phrase 696<br><br>S16 "quality of life" or "life quality" or HRQL or HRQOL or wellbeing or "well-being" or "well being" Search modes - Boolean/Phrase 246,342<br><br>S15 (MH "Quality of Life") Search modes - Boolean/Phrase 115,867<br><br>S14 "self care" or selfcare or "self management" or selfmanagement or "self treatment" or selftreatment Search modes - Boolean/Phrase 62,401<br><br>S13 (MH "Self Care") Search modes - Boolean/Phrase 42,229<br><br>S12 S7 AND S11 Search modes - Boolean/Phrase 636<br><br>S11 S8 OR S9 OR S10 Search modes - Boolean/Phrase 24,57<br><br>S10 ((video* or telephone* or tele* or digital or "e-mail" or email or remote) N2 (consult* or rehab* or counsel* or therap*)) Search modes - Boolean/Phrase 6,154<br><br>S9 telemedicine or teleconsult or telepatholog* or telerehab* or videoconsult* or skype or "Mobile Health" or ehealth or mhealth or "digital health" Search modes - Boolean/Phrase 19,5<br><br>S8 (MH "Telemedicine+") Search modes - Boolean/Phrase 13,227<br><br>S7 S3 OR S6 Search modes - Boolean/Phrase 115,206<br><br>S6 S4 OR S5 Search modes - Boolean/Phrase 65,638<br><br>S5 "heart failure*" or "heart backward failure*" or "cardiac failure*" or "cardiac backward failure*" or "cardiac incompetence*" or "cardiac insufficienc*" or "cardiac stand still" or "cardial decompensation*" or "cardial insufficienc*" or "heart insufficienc*" or

Literature Search - Documentation

	"insufficientia cardis" or "myocardial failure*" or "myocardial insufficienc*" Search modes - Boolean/Phrase 65,594<br><br>S4 (MH "Heart Failure+") Search modes - Boolean/Phrase 42,634<br><br>S3 S1 OR S2 Search modes - Boolean/Phrase 49,692<br><br>S2 ((colorectal or colon* or rectal or sigmoid or "sigmoid colon" or "colon sigmoid" or mesocolon) W0 (cancer* or carcinoma* or neoplasm* or tumor* or tumour*)) Search modes - Boolean/Phrase 48,046<br><br>S1 (MH "Colorectal Neoplasms+") Search modes - Boolean/Phrase 40,837
**Number of results**	135
**Comments**	

**Database/source**	Google Scholar
**Date of search**	23.03.2020
**Search History**	4 individual smaller searches carried out in order to get the articles with highest Google Page Rank:<br><br>Search 1:<br>"Heart failure" telehealth|telemedicine|mhealth|ehealth<br><br>Search 2:<br>"Colon cancer" telehealth|telemedicine|mhealth|ehealth<br><br>Search 3:<br>"Colorectal cancer" telehealth|telemedicine|mhealth|ehealth<br><br>Search 4<br>"Rectal cancer" telehealth|telemedicine|mhealth|ehealth
**Number of results**	200 (the first 50 articles (highest Page Rank) were selected for each search, totalling 200 articles. This was carried out using Harzing.com's Publish or Perish software.)
**Comments**	
